# Abdominal Compartment Syndrome: pathophysiology and definitions

**DOI:** 10.1186/1757-7241-17-10

**Published:** 2009-03-02

**Authors:** Michael L Cheatham

**Affiliations:** 1 Department of Surgical Education, Orlando Regional Medical Center, Orlando, Florida 32806, USA

## Abstract

"Intra-abdominal hypertension", the presence of elevated intra-abdominal pressure, and "abdominal compartment syndrome", the development of pressure-induced organ-dysfunction and failure, have been increasingly recognized over the past decade as causes of significant morbidity and mortality among critically ill surgical and medical patients. Elevated intra-abdominal pressure can cause significant impairment of cardiac, pulmonary, renal, gastrointestinal, hepatic, and central nervous system function. The significant prognostic value of elevated intra-abdominal pressure has prompted many intensive care units to adopt measurement of this physiologic parameter as a routine vital sign in patients at risk. A thorough understanding of the pathophysiologic implications of elevated intra-abdominal pressure is fundamental to 1) recognizing the presence of intra-abdominal hypertension and abdominal compartment syndrome, 2) effectively resuscitating patients afflicted by these potentially life-threatening diseases, and 3) preventing the development of intra-abdominal pressure-induced end-organ dysfunction and failure. The currently accepted consensus definitions surrounding the diagnosis and treatment of intra-abdominal hypertension and abdominal compartment syndrome are presented.

## Review

**Figure 1:**
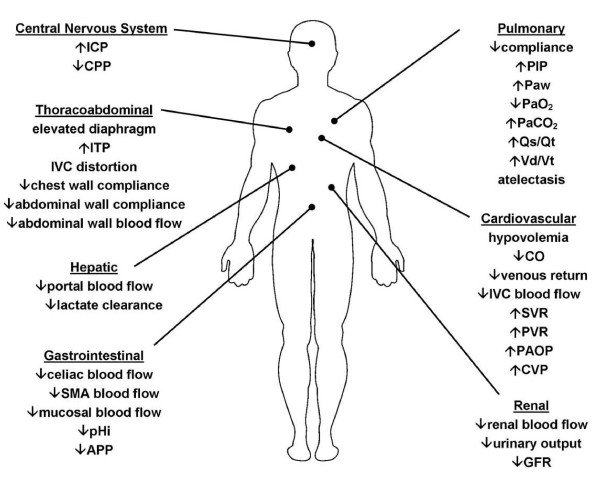
**Pathophysiologic Implications of Intra-abdominal Hypertension**. The effects of intra-abdominal hypertension are not limited just to the intra-abdominal organs, but rather have an impact either directly or indirectly on every organ system in the body. ICP – intracranial pressure; CPP – cerebral perfusion pressure; ITP – intrathoracic pressure; IVC – inferior vena cava; SMA – superior mesenteric artery; pHi – gastric intramuscosal pH; APP – abdominal perfusion pressure; PIP- peak inspiratory pressure; Paw – mean airway pressure; PaO_2 _– oxygen tension; PaCO_2 _– carbon dioxide tension; Qs/Qt – intrapulmonary shunt; Vd/Vt – pulmonary dead space ; CO – cardiac output; SVR – systemic vascular resistance; PVR – pulmonary vascular resistance; PAOP – pulmonary artery occlusion pressure; CVP – central venous pressure; GFR – glomerular filtration rate.

Although initially recognized over 150 years ago, the pathophysiologic implications of elevated intra-abdominal pressure (IAP) have essentially been rediscovered only within the past two decades [1-3]. An explosion of scientific investigation and accumulation of clinical experience has confirmed the significant detrimental impact of both "intra-abdominal hypertension" (IAH) (see figure 1), the presence of elevated intra-abdominal pressure, and "abdominal compartment syndrome" (ACS), the development of IAH-induced organ-dysfunction and failure, among the critically ill [4,5]. IAH has been identified as a continuum of pathophysiologic changes beginning with regional blood flow disturbances and culminating in frank end-organ failure and the development of ACS. ACS has been identified to be a cause of significant morbidity and mortality among critically ill surgical, medical, and pediatric patients. Previously present, but significantly under-appreciated, IAH and ACS are now recognized as common occurrences in the intensive care unit (ICU) setting [6-16]. Elevated IAP has been identified as an independent predictor of mortality during critical illness and likely plays a major role in the development of multiple system organ failure, a syndrome which has plagued ICU patients and physicians for decades [8,17,18].

Recently, evidence-based consensus definitions and recommendations for the resuscitation and rehabilitation of patients with IAH and ACS have been published [19,20]. Central to this evolving strategy are the use of early serial IAP measurements to detect the presence of IAH, application of comprehensive medical management strategies to reduce elevated IAP and restore end-organ perfusion, timely surgical abdominal decompression for refractory organ dysfunction, and early attempts at fascial closure once physiologically appropriate [21,22]. Such a strategy has been demonstrated to significantly improve patient survival, reduce complications (such as enteroatmospheric fistula), and decrease resource utilization [23,24]. The following review addresses both the pathophysiologic impact of elevated IAP on the various organ systems as well as the currently accepted definitions surrounding IAH and ACS. The diagnosis, prevention, and treatment of IAH/ACS have been addressed in a number of recent publications [6,10,12,13,19-22,24-29].

### History

The impact of elevated IAP upon respiratory function was first documented by Marey in 1863 and subsequently by Burt in 1870 [30]. In 1890, Henricius identified in an animal model that an IAP between 27 and 46 cm H_2_O significantly impaired diaphragmatic excursion leading to elevated intrathoracic pressure, respiratory failure, and death [30]. The theory that respiratory failure is the cause of death in severe IAH persisted until 1911 when Emerson demonstrated in cat, dog, and rabbit models that elevated IAP causes death by cardiovascular collapse rather than by respiratory failure [30]. The detrimental effect of elevated IAP on renal function and urinary output was first identified by Wendt in 1876 and the restoration of urinary output through abdominal decompression by Thorington and Schmidt in 1923 [31-33]. Overholt extensively studied the properties of the abdominal wall and confirmed that normal IAP is subatmospheric and that procedures which restrict movement of the abdominal wall or distention of the stomach or colon all result in an increase in IAP [34]. He postulated that IAP is governed by both the pressure induced by the abdominal contents and the "flexibility" (compliance) of the abdominal wall. Investigation into the physiologic effects of IAP on renal function in humans essentially began in 1947 with the work of Bradley [35]. The experiences of surgeons treating infants with gastroschisis or omphalocele further contributed to our understanding of both the concept of "loss of abdominal domain" as well as the life-threatening cardiac, pulmonary, and gastrointestinal complications which can occur when abdomens are primarily closed without consideration of elevated IAP [36-39]. Gross, in 1948, first described the use of a "staged abdominal repair" in the management of such infants unknowingly pioneering the open abdomen techniques which have now become standard in the treatment of IAH and ACS [36].

Although surrogate measurement of IAP via measurement of intravesicular, intragastric, and intracolonic pressure in animal models was commonplace in the 1920's and 1930's, it was Söderberg who, in 1970, first described the strong correlation between IAP and intravesicular pressure during laparoscopy in humans [40]. The landmark work of Harman, Kron, and Richards in the early 1980's "rediscovered" IAH as a cause of unexplained oliguria and subsequent renal failure in post-operative patients with abdominal distention [32,41,42]. They further reported the benefits of open abdominal decompression in restoring renal function and improving patient outcome in patients with an IAP in excess of 25 mmHg [32,41]. The introduction of laparoscopic techniques into mainstream surgical practice in the late 1980's and early 1990's led to numerous experimental and clinical studies which further advanced our understanding of the injurious effects of elevated IAP on cardiac, pulmonary, renal, gastrointestinal, hepatic, and cerebral function. Increased appreciation of these effects by both anesthesiologists and surgeons set the stage for recognition of both IAH and ACS in the critically ill patient population.

### Pathophysiology

An increasing body of literature has identified the significant physiologic derangements that occur as a result of elevated IAP. The effects of IAH are not limited just to the intra-abdominal organs, but rather have an impact either directly or indirectly on every organ system in the body. As a result, patients with prolonged, untreated IAH commonly manifest significant malperfusion and subsequent organ failure. Pre-existing comorbidities, such as chronic renal failure, pulmonary disease, or cardiomyopathy, play an important role in aggravating the effects of elevated IAP and may reduce the threshold of IAH that causes the clinical manifestations of ACS. The etiology for the patient's IAH is similarly of vital importance and may be determined as being either intra-abdominal, as occurs in surgical or trauma patients following damage control laparotomy, or extra-abdominal, as occurs in medical patients with sepsis or burn patients who require aggressive fluid resuscitation [6,7,43-46].

#### Cardiovascular

As originally described over 80 years ago by Emerson, rising IAP increases intrathoracic pressure through cephalad deviation of the diaphragm [30]. Increased intrathoracic pressure significantly reduces venous return resulting in reduced cardiac output [33,47-57]. Such reductions have been demonstrated to occur at an IAP of only 10 mmHg [18,57]. Hypovolemic patients appear to sustain reductions in cardiac output at lower levels of IAP than do normovolemic patients [50,53]. Hypervolemic patients demonstrate increased venous return in the presence of mild to moderate elevations in IAP suggesting that volume resuscitation may have a protective effect [53]. Diaphragmatic elevation and increased intrathoracic pressure have also been postulated to cause direct cardiac compression reducing ventricular compliance and contractility [49]. Systemic vascular resistance (afterload) is increased through compression of both the aorta and systemic vasculature and pulmonary vascular resistance through compression of the pulmonary parenchyma [33,48,51-56,58]. As a result, in the absence of severe IAH, mean arterial pressure typically remains stable despite a decrease in venous return and cardiac output. Such increases in afterload may be poorly tolerated by those with marginal cardiac contractility or inadequate intravascular volume. Preload augmentation through volume administration appears to ameliorate, at least partially, the injurious effects of IAH-induced increases in afterload [18,33,48,53,56,58,59].

Paradoxically, intracardiac filling pressures such as pulmonary artery occlusion ("wedge") pressure (PAOP) and central venous pressure (CVP) typically increase with rising IAP despite the reduced venous return and cardiac output [47-49,51,53,56,57,59-64]. This apparent deviation from Starling's Law of the heart is due to the fact that both PAOP and CVP are measured relative to atmospheric pressure and are actually the sum of both intravascular pressure and intrathoracic pressure [63,64]. In the presence of IAH-induced elevations in intrathoracic pressure, PAOP and CVP tend to be erroneously elevated and no longer reflective of true intravascular volume status [47-49,57,59-61,63,64]. Such alterations in PAOP and CVP have been demonstrated with an IAP of only 10 mmHg [57]. Attempts to correct for this measurement error through use of transmural pressures (i.e., PAOP minus intrathoracic pressure) has confirmed that transmural PAOP decreases with rising IAP correctly reflecting the decreased venous return and cardiac preload [59]. Several studies have demonstrated that volumetric parameters, such as right ventricular end-diastolic volume (RVEDV), global end-diastolic volume (GEDV), or stroke volume variation (SVV) are superior predictors of intravascular volume status whose accuracy is unaffected by changes in intrathoracic pressure [63-66]. When traditional intracardiac filling pressures must be used, transmural pressures may be estimated as follows [63,64]:

Transmural PAOP = PAOP - 0.5*IAP

Transmural CVP = CVP - 0.5*IAP

IAH also reduces venous return from the lower extremities functionally obstructing inferior vena caval blood flow by two mechanisms. First, inferior vena caval pressure increases significantly in the presence of IAH and has been demonstrated to parallel changes in IAP [18,33,53,56]. Second, cephalad deviation of the diaphragm causes a mechanical narrowing of the vena cava at the diaphragmatic crura further reducing venous return to the heart [54,67]. Femoral vein pressures are markedly increased and venous blood flow and pulsatility dramatically reduced [68,69]. The resulting increases in extremity venous hydrostatic pressure promote the formation of peripheral edema. These changes place the patient with IAH at risk for development of deep venous thrombosis [69-71]. Reduction of IAP restores femoral venous blood flow, but has anecdotally been reported to result in pulmonary embolism [71].

#### Pulmonary

The pulmonary effects of elevated IAP have been recognized for many years [30,33,49,51,59,68,72-74]. IAP is transmitted to the thorax both directly and through cephalad deviation of the diaphragm. This significantly increases intrathoracic pressure resulting in extrinsic compression of the pulmonary parenchyma and development of pulmonary dysfunction [18,47,48,57,59,68]. Compression of the pulmonary parenchyma appears to begin with an IAP of 16–30 mmHg and is accentuated by the presence of hemorrhagic shock and hypotension [57,75]. Parenchymal compression results in alveolar atelectasis, decreased oxygen transport across the pulmonary capillary membrane, and an increased intrapulmonary shunt fraction (Qsp/Qt). IAH-induced atelectasis has been demonstrated to cause an increase in the rate of pulmonary infection [76]. Parenchymal compression also reduces pulmonary capillary blood flow leading to decreased carbon dioxide excretion and an increased alveolar dead space (Vd/Vt) [57]. Both peak inspiratory and mean airway pressures are significantly increased and may result in alveolar volutrauma [57,75]. Spontaneous tidal volumes and dynamic pulmonary compliance are reduced resulting in further ventilation-perfusion mismatching [57,75]. In combination, these effects lead to the arterial hypoxemia and hypercarbia that, in part, characterize ACS [18,33,48,51,59,73].

#### Renal

IAH-induced reductions in renal blood flow and function have been demonstrated in both animal and human models [33,35,42,51,77]. These changes occur in direct response to increasing IAP with oliguria developing at an IAP of 15 mmHg and anuria at 30 mmHg [32,33,42]. Renal artery blood flow has been demonstrated to be preferentially diminished in comparison to both celiac and superior mesenteric artery blood flow [68]. Renal vein pressure and renal vascular resistance are both significantly elevated [35,42,48]. All of these changes shunt blood away from the renal cortex and functioning glomeruli leading to impaired glomerular and tubular function and significant reductions in urinary output [32,33,35,41,42,48,49,51,73,77-80].

Several mechanisms have been proposed as the etiology for IAH-induced renal dysfunction and failure. Harman et al. negated direct ureteral compression as a cause through studies utilizing ureteral stents [42]. Other authors have suggested that direct parenchymal compression and development of a "renal compartment syndrome" results in renal ischemia and subsequent failure [70,81]. Stone demonstrated in traumatically injured patients that incising the renal capsule could reverse renal failure if performed early and prior to development of severe renal dysfunction [81]. Recent studies suggest that compression of the renal vein likely plays the primary role in the development of renal dysfunction with reduced cardiac output playing a secondary role [32,33,48,81].

IAH decreases glomerular filtration rate causing a rise in both blood urea nitrogen and serum creatinine and a reduction in creatinine clearance [33,35,42,48,51,79]. Osmolar clearance is similarly decreased and fractional excretion of sodium increased [79]. Urinary sodium and chloride concentrations decrease and urinary potassium concentrations increase [33]. Plasma renin activity and aldosterone levels increase significantly [33,48]. Antidiuretic hormone levels have been demonstrated to increase to more than twice basal levels [82]. All of these pathophysiologic changes appear to be potentially reversible if the patient's IAH is recognized and treated appropriately before significant organ dysfunction has developed [32,48].

#### Gastrointestinal

Of all the organ systems, the gut appears to be one of the most sensitive to elevations in IAP. Such reductions in mesenteric blood flow may appear with an IAP of only 10 mmHg [83]. Caldwell et al. has demonstrated decreased blood flow to virtually all intra-abdominal and retroperitoneal organs as a result of elevated IAP [56]. The sole exception was adrenal blood flow which appears to be preserved and has been postulated to be a survival mechanism by which to support catecholamine release in the face of ongoing shock [56]. Celiac artery blood flow is reduced by up to 43% and superior mesenteric artery blood flow by as much as 69% in the presence of intra-abdominal pressures of 40 mmHg [68,83,84]. The negative effects of IAP on mesenteric perfusion are augmented by the presence of hypovolemia or hemorrhage [8,50,68,83,85]. Reintam et al. have recently validated a grading system for predicting mortality due to gastrointestinal dysfunction among patients with IAH/ACS [86].

In addition to reducing arterial blood flow, IAP compresses thin walled mesenteric veins promoting venous hypertension and intestinal edema. Visceral swelling further increases IAP initiating a vicious cycle which results in worsening malperfusion, bowel ischemia, decreased intramucosal pH, feeding intolerance, systemic metabolic acidosis, and significantly increased patient mortality [8,13,50,86,87]. Intestinal mucosal perfusion is diminished by levels of IAP as low as 20 mmHg as demonstrated using gastric or colonic tonometry and by laser flow probe [8,50,84,87]. Sugrue et al. found that patients with IAH were over 11 times more likely to have abnormal gastric intramucosal pH measurements than were those without IAH [87]. Djavani et al have recently reported a similar significant correlation between abnormal colonic intramucosal pH and IAH [85]. They have further confirmed a high risk of colonic ischemia in post-abdominal aortic aneurysmectomy patients with IAP > 20 mmHg [88]. Malperfusion of the gut as a result of elevated IAP has been speculated as a possible mechanism for loss of the mucosal barrier and subsequent development of bacterial translocation, sepsis, and multiple system organ failure [84,89,90]. Gargiulo et al. demonstrated bacterial translocation to mesenteric lymph nodes in the presence of hemorrhage and an IAP of only 10 mmHg [90].

#### Hepatic

Hepatic artery, hepatic vein, and portal vein blood flow are all reduced by the presence of IAH [50,52,54,77,91]. Hepatic artery flow is directly affected by decreases in cardiac output. Hepatic and portal venous flow are diminished as a result of both extrinsic compression of the liver as well as anatomic narrowing of the hepatic veins as they pass through the diaphragm [67]. Increased hepatic vein pressures have been demonstrated to result in increased azygos vein blood flow suggesting a compensatory increase in gastroesophageal collateral blood flow in response to hepatic venous congestion [54]. On a microscopic level, hepatic microcirculatory blood flow is decreased resulting in a reduction in hepatic mitochondrial function and production of energy substrates [50,91]. Lactic acid clearance by the liver appears to be compromised potentially confounding its use as a marker of resuscitation adequacy [92]. Of particular importance is that these changes have been documented with IAP elevations of only 10 mmHg and in the presence of both normal cardiac output and mean arterial blood pressure [50].

#### Central Nervous System

Cerebral perfusion and function are also directly affected by the presence of IAH. According to the Monroe-Kellie doctrine, the brain consists of four discrete compartments: parenchymal, vascular, osseous, and cerebrospinal fluid. An increase in the pressure within one compartment results in a reciprocal increase in the pressure within each of the other non-osseous compartments. Whereas chronic, slowly developing increases in intracranial pressure (ICP) may allow time for compensation, the acute increases in ICP characteristic of both traumatic injury and acute illness commonly result in rapidly escalating intracranial pressures. Elevations in intra-abdominal and intrathoracic pressure may also directly impact the pressures within the cranium. Coughing, defecating, emesis, and other common causes of increased intra-abdominal and intrathoracic pressure are well known to transiently increase ICP [48,93,94]. IAH can induce similar increases in ICP, but these elevations are sustained as long as the IAH is present and can result in significant reductions in cerebral perfusion pressure (CPP) [47,48,61,94-96]. The mechanism by which IAH causes elevations in ICP has long been a subject of debate [47,48,94,97,98]. Proposed mechanisms have included decreased lumbar venous plexus blood flow (leading to increased CSF pressure), increased PaCO_2 _(resulting in increased cerebral blood flow), and decreased cerebral venous outflow [47,48,94,97,98]. Luce et al. in a series of animal experiments and Bloomfield et al. in clinical studies involving humans have confirmed that increased intrathoracic pressure impairs venous return from the cranium and decreases cerebral venous blood flow [48,97]. This increases intracranial venous blood volume in a manner similar to that encountered with the use of both PEEP and military anti-shock trousers [97-99]. Intracerebral venous pooling can markedly worsen pre-existing cerebral perfusion abnormalities due to trauma, chronic intracranial hypertension, or other causes of decreased cerebral compliance [96,98]. Sugerman et al. have demonstrated that normal cerebral compliance appears to be protective against intrathoracic pressure-induced increases in ICP [96]. Decreased pulmonary compliance as a result of severe pulmonary dysfunction, as occurs in IAH, also appears to have a protective effect on ICP [61,95]. Hypovolemia, on the other hand, may worsen already marginal cerebral perfusion [79,95].

#### Abdominal wall

Although commonly overlooked, the abdominal wall is also subject to the effects of elevated IAP. Visceral edema, abdominal packs, and free intraperitoneal fluid all distend the abdomen and reduce abdominal wall compliance [67,100]. Abdominal wall edema secondary to shock and fluid resuscitation also decreases abdominal compliance. Previous pregnancy, morbid obesity, cirrhosis, and other conditions associated with increased abdominal wall compliance all appear to be protective, to an extent, against the development of IAH [87,96]. Diebel et al. have demonstrated that IAH dramatically reduces abdominal wall blood flow [101]. Rectus sheath blood flow decreases to 58% of baseline at an IAP of only 10 mmHg and to 20% of baseline at 40 mmHg [101]. These findings may explain the impaired wound healing, high rate of fascial dehiscence, and predilection to development of necrotizing fasciitis identified in patients whose abdomens are closed under tension [70,101].

### Definitions

In 2004, a consensus conference was convened by the World Society of the Abdominal Compartment Syndrome (WSACS)  consisting of European, Australasian, and North American surgical, trauma, and medical critical care specialists. Recognizing the lack of accepted definitions, and the resulting confusion and difficulty in comparing studies published in this area, the WSACS tasked these specialists to create evidence-based definitions for IAH and ACS. After extensively reviewing the existing literature, the authors suggested a conceptual framework for standardizing the definitions of IAH and ACS as well as a general technique for IAP monitoring based upon the current understanding of the pathophysiology of these two syndromes [19]. A brief summary of these definitions follows (Table 1).

**Table 1:** Definitions

Definition 1	IAP is the steady-state pressure concealed within the abdominal cavity.
Definition 2	APP = MAP - IAP

Definition 3	FG = GFP - PTP = MAP - 2 * IAP

Definition 4	IAP should be expressed in mmHg and measured at end-expiration in the complete supine position after ensuring that abdominal muscle contractions are absent and with the transducer zeroed at the level of the mid-axillary line.

Definition 5	The reference standard for intermittent IAP measurement is via the bladder with a maximal instillation volume of 25 mL of sterile saline.

Definition 6	Normal IAP is approximately 5–7 mmHg in critically ill adults.

Definition 7	IAH is defined by a sustained or repeated pathologic elevation of IAP ≥ 12 mmHg.

Definition 8	IAH is graded as follows:
	• Grade I: IAP 12–15 mmHg
	• Grade II: IAP 16–20 mmHg
	• Grade III: IAP 21–25 mmHg
	• Grade IV: IAP > 25 mmHg

Definition 9	ACS is defined as a sustained IAP > 20 mmHg (with or without an APP < 60 mmHg) that is associated with new organ dysfunction/failure.

Definition 10	Primary ACS is a condition associated with injury or disease in the abdomino-pelvic region that frequently requires early surgical or interventional radiological intervention.

Definition 11	Secondary ACS refers to conditions that do not originate from the abdomino-pelvic region.

Definition 12	Recurrent ACS refers to the condition in which ACS redevelops following previous surgical or medical treatment of primary or secondary ACS.

Consensus definitions as proposed by the International Conference of Experts on Intra-abdominal Hypertension and Abdominal Compartment Syndrome.

#### Intra-abdominal pressure (IAP)

The abdomen may be considered as a closed box with walls that are either rigid (costal arch, spine, and pelvis) or flexible (abdominal wall and diaphragm). The compliance of these walls and the volume of the organs contained within determine the pressure within the abdomen at any given time [102-104] IAP is defined as the steady-state pressure concealed within the abdominal cavity, increasing with inspiration (diaphragmatic contraction) and decreasing with expiration (diaphragmatic relaxation). IAP is directly affected by the volume of the solid organs or hollow viscera (which may be either empty or filled with air, liquid or fecal matter), the presence of ascites, blood or other space-occupying lesions (such as tumors or a gravid uterus), and the presence of conditions that limit expansion of the abdominal wall (such as burn eschars or third-space edema) [19].

#### Abdominal perfusion pressure (APP)

Analogous to the widely utilized concept of cerebral perfusion pressure, abdominal perfusion pressure (APP), defined as MAP minus IAP, has been demonstrated to be an accurate predictor of visceral perfusion and an endpoint for resuscitation [64,105,106]. APP, by considering both arterial inflow (MAP) and restrictions to venous outflow (IAP), is statistically superior to either parameter alone in predicting patient survival from IAH and ACS [64,105,106]. APP is also superior to other common resuscitation endpoints such as arterial pH, base deficit, arterial lactate, and hourly urinary output. Failure to maintain an APP of at least 60 mmHg by day 3 of critical illness has been demonstrated to predict survival from IAH and ACS [64,105,106]. APP thus figures prominently in the resuscitation strategy recommended by the WSACS.

#### Filtration Gradient

As described above, oliguria is one of the first visible signs of IAH. Inadequate renal perfusion pressure and renal filtration gradient (FG) have been proposed as key factors in the development of IAP-induced renal failure [107,108]. The FG is the mechanical force across the glomerulus and equals the difference between the glomerular filtration pressure (GFP) and the proximal tubular pressure (PTP). In the presence of IAH, GFP may be approximated as MAP minus IAP (or APP) while PTP may be assumed to equal IAP. The FG is thus defined as MAP minus two times the IAP, illustrating that changes in IAP have a greater impact upon renal function and urine production than do changes in MAP.

#### IAP measurement

The sensitivity of both clinical judgement and physical examination have been demonstrated to be very poor in predicting a patient's IAP [109,110]. Early, serial IAP measurements are therefore essential to both diagnosing the presence of IAH as well as guiding resuscitative therapy [111]. While a variety of methods for IAP measurement have been described, intravesicular or "bladder" pressure has achieved the most widespread adoption worldwide due to its simplicity, minimal cost, and low risk of complications [103,112-115]. Several key points must be considered to ensure accurate and reproducible IAP measurements. Early IAH studies utilized water manometers to determine IAP with results reported in cm H_2_O while subsequent studies using electronic pressure transducers reported IAP in mmHg (1 mmHg = 1.36 cm H_2_O). This led to confusion and difficulty in comparing studies. A point of further confusion has been the appropriate zero reference point for the abdomen. Changes in body position (i.e., supine, prone, head of bed elevated) can have a significant impact upon the measured IAP. While head of bed elevation is now commonly performed to reduce the incidence of ventilator-associated pneumonia, the clinical studies that determined the threshold IAP values that lead to organ dysfunction were determined in the supine position. Further, the presence of both abdominal and bladder detrusor muscle contractions have been demonstrated to impact the accuracy of IAP measurements. Perhaps the greatest point of contention has been the proper priming-volume to be instilled into the bladder to ensure a conductive fluid column between bladder wall and transducer. Large instillation volumes, as commonly utilized in years past, have been demonstrated to result in artificial increases in IAP that could lead to inappropriate therapy. In an attempt to address these issues and ensure both the accuracy and reproducibility of IAP measurements, the WSACS has recommended that IAP be expressed in mmHg and measured at end-expiration in the complete supine position after ensuring that abdominal muscle contractions are absent and with the transducer zeroed at the level of the mid-axillary line [20]. Further, IAP should be measured via the bladder with a maximal instillation volume of 25 mL of sterile saline [20].

#### Normal and Pathologic IAP values

Normal IAP ranges from sub-atmospheric to zero mmHg [109,113,116]. In the typical intensive care unit patient, however, IAP is commonly elevated to a range of 5–7 mmHg while patients with recent abdominal surgery, sepsis, organ failure, or need for volume resuscitation may demonstrate IAPs of 10–20 mmHg [11,15]. Prolonged elevation in IAP to such levels can result in organ dysfunction and failure while pressures above 25 mmHg are associated with significant potential mortality [65,80,105].

#### Intra-Abdominal Hypertension (IAH)

Pathological IAP is a continuum ranging from mild IAP elevations without clinically significant adverse effects to substantial increases in IAP with grave consequences to virtually all organ systems in the body. The exact IAP that defines IAH has long been debated. Burch et al. defined an early grading system for IAH (in cm H_2_O) as follows: Grade I, 7.5–11 mmHg (10–15 cm H_2_0); Grade II, 11–18 mmHg (15–25 cm H_2_0); Grade III, 18–25 mmHg (25–35 cm H_2_0); and Grade IV, > 25 mmHg (> 35 cm H_2_0) [117]. Burch suggested that most patients with Grade III and all patients with Grade IV should undergo abdominal decompression. The deleterious effects of elevated IAP on renal, cardiac, and gastrointestinal function, however, may be witnessed at IAP levels as low as 10–15 mmHg which would be classified as Grade I in the Burch system [11,44,87,104,118-124]. In recognition of the pathophysiologic impact of these lower levels of IAP, the WSACS has defined IAH as a sustained or repeated pathologic elevation of IAP ≥ 12 mmHg. The WSACS has also modified the Burch system to increase its clinical sensitivity as follows: Grade I: IAP 12–15 mmHg; Grade II: IAP 16–20 mmHg; Grade III: IAP 21–25 mmHg; and Grade IV: IAP > 25 mmHg [19,20]. In this scenario, medical intervention is appropriate for any grade of IAH while surgical decompression is typically reserved for Grade IV IAH.

#### Abdominal compartment syndrome (ACS)

Among the majority of patients, critical IAP appears to be 10–15 mmHg. It is at this pressure that reductions in microcirculatory blood flow occur and the initial signs of organ dysfunction and failure are witnessed. ACS is the natural progression of these pressure-induced end-organ changes and develops if IAH is not recognized and treated in a timely manner. Failure to recognize and appropriately treat ACS is commonly fatal while prevention and/or timely intervention is associated with marked improvements in organ function and patient survival [8,11,23,44,125-127].

In contrast to IAH, ACS is not graded, but rather considered an "all or nothing" phenomenon. The WSACS defines ACS as a sustained IAP > 20 mmHg (with or without an APP < 60 mmHg) that is associated with new organ dysfunction or failure (Appendix 1) [19,20]. ACS may be further classified as either primary, secondary, or recurrent based upon the duration and etiology of the patient's IAH. Primary ACS is characterized by IAH of relatively brief duration occurring as a result of an intra-abdominal etiology such as abdominal trauma, ruptured abdominal aortic aneurysm, hemoperitoneum, acute pancreatitis, secondary peritonitis, retroperitoneal haemorrhage, or liver transplantation. Primary ACS is therefore defined as a condition associated with injury or disease in the abdomino-pelvic region that frequently requires early surgical or interventional radiological intervention. It is most commonly encountered in the traumatically injured or post-operative surgical patient. Secondary ACS is characterized by IAH that develops as a result of an extra-abdominal etiology such as sepsis, capillary leak, major burns, or other conditions requiring massive fluid resuscitation. It is most commonly encountered in the medical or burn patient [43,104,128,129]. Recurrent ACS represents a redevelopment of ACS symptoms following resolution of an earlier episode of either primary or secondary ACS. It is most commonly associated with the development of acute IAH in a patient who is recovering from IAH/ACS and therefore represents a "second-hit" phenomenon. It may occur despite the presence of an open abdomen or as a new ACS episode following definitive closure of the abdominal wall. Recurrent ACS, due to the patient's current or recent critical illness, is associated with significant morbidity and mortality.

## Conclusion

Elevated IAP commonly causes marked deficits in both regional and global perfusion that, when unrecognized, result in significant organ failure and patient morbidity and mortality. Significant progress has been made over the past decade with regard to understanding the etiology of IAH and ACS as well as implementing appropriate resuscitative therapy. Routine measurement of IAP in patients at risk is essential to both recognizing the presence of IAH/ACS and guiding effective treatment. Adoption of the proposed consensus definitions and recommendations has been demonstrated to significantly improve patient survival from IAH/ACS and will facilitate future investigation in this area.

## Abbreviations

IAP: intra-abdominal pressure; IAH: intra-abdominal hypertension; ACS: abdominal compartment syndrome; MAP: mean arterial pressure; APP: abdominal perfusion pressure; FG: filtration gradient; GFP: glomerular filtration pressure; PTP: proximal tubular pressure; PIP: peak inspiratory pressure; FiO_2_: fraction of inspired oxygen; PEEP: positive end-expiratory pressure; ICP: intracranial pressure; PAOP: pulmonary artery occlusion pressure; CVP: central venous pressure.

## Competing interests

Financial competing interests

• Dr. Cheatham has served as a consultant for Kinetic Concepts, Inc., Wolfe-Tory Medical, Inc., and Bard Medical, Inc.

Non-financial competing interests

• Dr. Cheatham is a member of the World Society of the Abdominal Compartment Syndrome Executive Committee.

## Authors' contributions

MLC is the sole contributor to this manuscript.

## Appendix 1 – Signs of Abdominal Compartment Syndrome

Abdominal distention

Elevated IAP

Oliguria refractory to volume administration

Elevated PIP

Hypercarbia

Hypoxemia refractory to increasing FiO2 and PEEP

Refractory metabolic acidosis

Elevated ICP

Legend: These represent the most common organ dysfunctions associated with the development of severe intra-abdominal hypertension and a diagnosis of abdominal compartment syndrome.
